# 1,3-Bis(1-adamant­yl)imidazolium tetra­chloridoferrate(III)

**DOI:** 10.1107/S1600536810043989

**Published:** 2010-10-31

**Authors:** Monisola I. Ikhile, Muhammad D. Bala

**Affiliations:** a School of Chemistry, University of KwaZulu-Natal, Westville Campus, Private Bag X54001, Durban 4000, South Africa

## Abstract

The crystal structure of the title compound, (C_23_H_33_N_2_)[FeCl_4_], consists of 1,3-bis­(1-adamant­yl)imidazolium (BAIM) cations and tetra­hedral tetra­chloridoferrate(III) (TCF) anions. The BAIM cation possesses *m* symmetry, with the central imidazole ring and four C atoms of each terminal adamantyl group located on a mirror plane. The Fe and two Cl atoms of the TCF anion are also located on the mirror plane. The cyclo­hexane rings of the adamantyl groups adopt normal chair conformations.

## Related literature

For related structures based on the 1,3-bis­(adamant­yl)­imidazolium unit, see: Grossie *et al.* (2006 ▶, 2009 ▶). For a related synthetic procedure, see: Louie & Grubbs (2000 ▶). For related *N*-heterocyclic carbene structures in general, see: Arduengo *et al.* (1991 ▶).
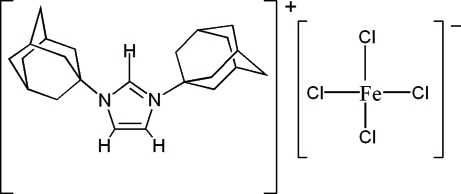


## Experimental

### 

#### Crystal data

(C_23_H_33_N_2_)[FeCl_4_]
*M* *_r_* = 535.16
Orthorhombic, 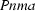

*a* = 15.3517 (4) Å
*b* = 9.7557 (3) Å
*c* = 16.3502 (4) Å
*V* = 2448.71 (12) Å^3^
*Z* = 4
Mo *K*α radiation
μ = 1.07 mm^−1^
*T* = 173 K
0.29 × 0.22 × 0.20 mm

#### Data collection

Bruker APEXII CCD diffractometer
Absorption correction: multi-scan (*SADABS*; Sheldrick, 1996 ▶) *T*_min_ = 0.747, *T*_max_ = 0.815
14913 measured reflections
3126 independent reflections
2410 reflections with *I* > 2σ(*I*)
*R*_int_ = 0.049

#### Refinement

*R*[*F* ^2^ > 2σ(*F* ^2^)] = 0.034
*wR*(*F* ^2^) = 0.089
*S* = 1.02
3126 reflections
160 parameters
H-atom parameters constrained
Δρ_max_ = 0.76 e Å^−3^
Δρ_min_ = −0.42 e Å^−3^

### 

Data collection: *APEX2* (Bruker, 2005 ▶); cell refinement: *SAINT-Plus* (Bruker, 2005 ▶); data reduction: *SAINT-Plus*; program(s) used to solve structure: *SHELXTL* (Sheldrick, 2008 ▶); program(s) used to refine structure: *SHELXTL*; molecular graphics: *PLATON* (Spek, 2009 ▶) and *ORTEP-3* (Farrugia, 1997 ▶); software used to prepare material for publication: *SHELXTL*.

## Supplementary Material

Crystal structure: contains datablocks global, I. DOI: 10.1107/S1600536810043989/xu5064sup1.cif

Structure factors: contains datablocks I. DOI: 10.1107/S1600536810043989/xu5064Isup2.hkl

Additional supplementary materials:  crystallographic information; 3D view; checkCIF report

## Figures and Tables

**Fig. 1.**
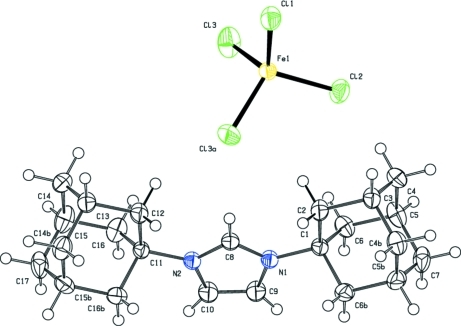
Molecular structure of the title complex with the atom labelling scheme. Ellipsoids are drawn at the 50% probability level.

## supplementary crystallographic information

### Comment

The title compound (I) was obtained in an attempt to couple the *N*-heterocyclic carbene (NHC) ligand to FeCl_2_ using the free carbene method. The anticipated coordination product was not obtained but a co-crystal of the ligand and FeCl_4_ anion was isolated as (I). Protonation of the NHC ligand and oxidation of the metal source observed in this process is of structural and synthetic interest because the free carbene method is commonly used for the preparation of NHC-metal complexes especially those supported by sterically demanding imidazolium salts. The structure of (I) is characterized by a symmetrical imidazolium unit and a tetrahedral iron cenre with the asymmetric unit containing an independent protonated 1,3 bis(adamantyl)imidazol-2-ylidene moiety and the tetrahedral tetrachloridoferrate(III) anion [FeCl_4_^-^]. The imidazolium moiety and the FeC*L*_4_^-^ anion are held together by a network of Cl(1)··· H(9) short contacts measured to be 2.904 (2) Å. In addition the molecule of (I) has a crystallographically imposed centrosymmetry and the imidazolium ring is completely planar. The cyclohexane groups of the adamantyl ligands adopt chair conformations.

### Experimental

1,3-Bis(adamantyl)imidazol-2-ylidenium chloride (0.1 g) and potassium *tert*-butoxide (0.04 g) were dissolved in 20 ml of THF and stirred at room temperature for 30 min. After evaporating the solvent, the free carbene was extracted in warm toluene (2 *x* 20 ml). This was followed by addition of 0.034 g of FeCl_2_ to the toluene solution and refluxed for 24 h. After removal of all volatiles, the residue was purified by recrystallization from dichloromethane/hexane to give X-ray quality orange block crystals of (I).

### Refinement

All H atoms attached to C atoms were fixed geometrically and treated as riding with C—H = 0.95-1.00 Å and *U*_iso_(H) = 1.2*U*_eq_(C).

### Crystal data

**Table:**

(C_23_H_33_N_2_)[FeCl_4_]	*F*(000) = 1116
*M**_r_* = 535.16	*D*_x_ = 1.452 Mg m^−^^3^
Orthorhombic, *P**n**m**a*	Mo *K*α radiation, λ = 0.71073 Å
Hall symbol: -P 2ac 2n	Cell parameters from 4384 reflections
*a* = 15.3517 (4) Å	θ = 2.5–28.1°
*b* = 9.7557 (3) Å	µ = 1.07 mm^−^^1^
*c* = 16.3502 (4) Å	*T* = 173 K
*V* = 2448.71 (12) Å^3^	Block, orange
*Z* = 4	0.29 × 0.22 × 0.20 mm

### Data collection

**Table:**

Bruker APEXII CCD diffractometer	3126 independent reflections
Radiation source: fine-focus sealed tube	2410 reflections with *I* > 2σ(*I*)
graphite	*R*_int_ = 0.049
φ and ω scans	θ_max_ = 28.0°, θ_min_ = 1.8°
Absorption correction: multi-scan (*SADABS*; Sheldrick, 1996)	*h* = −20→8
*T*_min_ = 0.747, *T*_max_ = 0.815	*k* = −12→9
14913 measured reflections	*l* = −21→20

### Refinement

**Table:**

Refinement on *F*^2^	Primary atom site location: structure-invariant direct methods
Least-squares matrix: full	Secondary atom site location: difference Fourier map
*R*[*F*^2^ > 2σ(*F*^2^)] = 0.034	Hydrogen site location: inferred from neighbouring sites
*wR*(*F*^2^) = 0.089	H-atom parameters constrained
*S* = 1.02	*w* = 1/[σ^2^(*F*_o_^2^) + (0.0488*P*)^2^] where *P* = (*F*_o_^2^ + 2*F*_c_^2^)/3
3126 reflections	(Δ/σ)_max_ = 0.001
160 parameters	Δρ_max_ = 0.76 e Å^−^^3^
0 restraints	Δρ_min_ = −0.42 e Å^−^^3^

### Special details

**Table:**

Geometry. All e.s.d.'s (except the e.s.d. in the dihedral angle between two l.s. planes) are estimated using the full covariance matrix. The cell e.s.d.'s are taken into account individually in the estimation of e.s.d.'s in distances, angles and torsion angles; correlations between e.s.d.'s in cell parameters are only used when they are defined by crystal symmetry. An approximate (isotropic) treatment of cell e.s.d.'s is used for estimating e.s.d.'s involving l.s. planes.
Refinement. Refinement of *F*^2^ against ALL reflections. The weighted *R*-factor *wR* and goodness of fit *S* are based on *F*^2^, conventional *R*-factors *R* are based on *F*, with *F* set to zero for negative *F*^2^. The threshold expression of *F*^2^ > σ(*F*^2^) is used only for calculating *R*-factors(gt) *etc*. and is not relevant to the choice of reflections for refinement. *R*-factors based on *F*^2^ are statistically about twice as large as those based on *F*, and *R*- factors based on ALL data will be even larger.

### Fractional atomic coordinates and isotropic or equivalent isotropic displacement parameters (Å^2^)

**Table:**

	*x*	*y*	*z*	*U*_iso_*/*U*_eq_	Occ. (<1)
C1	0.25910 (14)	0.2500	0.44556 (14)	0.0244 (5)	
C2	0.32128 (15)	0.2500	0.51793 (15)	0.0319 (6)	
H2A	0.3111	0.1677	0.5521	0.038*	0.50
H2B	0.3111	0.3323	0.5521	0.038*	0.50
C3	0.41569 (15)	0.2500	0.48571 (15)	0.0317 (6)	
H3	0.4570	0.2500	0.5330	0.038*	
C4	0.43059 (11)	0.1222 (2)	0.43410 (12)	0.0367 (4)	
H4A	0.4211	0.0393	0.4678	0.044*	
H4B	0.4914	0.1208	0.4138	0.044*	
C5	0.36751 (12)	0.1227 (2)	0.36191 (12)	0.0385 (5)	
H5	0.3778	0.0393	0.3276	0.046*	
C6	0.27317 (11)	0.1213 (2)	0.39388 (11)	0.0320 (4)	
H6A	0.2318	0.1201	0.3474	0.038*	
H6B	0.2631	0.0383	0.4274	0.038*	
C7	0.38239 (17)	0.2500	0.31053 (16)	0.0434 (7)	
H7A	0.4427	0.2500	0.2891	0.052*	
H7B	0.3419	0.2500	0.2634	0.052*	
C8	0.14111 (15)	0.2500	0.55290 (14)	0.0249 (5)	
H8	0.1788	0.2500	0.5990	0.030*	
C9	0.09341 (16)	0.2500	0.42699 (16)	0.0323 (6)	
H9	0.0922	0.2500	0.3689	0.039*	
C10	0.02372 (16)	0.2500	0.47676 (15)	0.0332 (6)	
H10	−0.0356	0.2500	0.4603	0.040*	
C11	−0.00008 (14)	0.2500	0.63217 (14)	0.0239 (5)	
C12	0.05956 (15)	0.2500	0.70717 (14)	0.0311 (6)	
H12A	0.0973	0.3323	0.7064	0.037*	0.50
H12B	0.0973	0.1677	0.7064	0.037*	0.50
C13	0.00332 (15)	0.2500	0.78484 (15)	0.0315 (6)	
H13	0.0419	0.2500	0.8341	0.038*	
C14	−0.05333 (13)	0.1222 (2)	0.78560 (11)	0.0366 (4)	
H14A	−0.0160	0.0394	0.7849	0.044*	
H14B	−0.0889	0.1202	0.8361	0.044*	
C15	−0.11249 (13)	0.1224 (2)	0.71109 (12)	0.0405 (5)	
H15	−0.1502	0.0388	0.7121	0.049*	
C16	−0.05666 (12)	0.1214 (2)	0.63305 (11)	0.0355 (4)	
H16A	−0.0194	0.0386	0.6320	0.043*	
H16B	−0.0947	0.1198	0.5842	0.043*	
C17	−0.16995 (16)	0.2500	0.71207 (17)	0.0458 (8)	
H17A	−0.2087	0.2500	0.6637	0.055*	
H17B	−0.2068	0.2500	0.7618	0.055*	
N1	0.16675 (12)	0.2500	0.47532 (12)	0.0253 (4)	
N2	0.05441 (12)	0.2500	0.55629 (12)	0.0249 (4)	
Cl1	0.63957 (4)	0.2500	0.30305 (4)	0.04157 (19)	
Cl2	0.66517 (5)	0.2500	0.52085 (4)	0.04157 (18)	
Cl3	0.81788 (4)	0.06894 (6)	0.39623 (4)	0.05261 (17)	
Fe1	0.73335 (2)	0.2500	0.40369 (2)	0.02584 (11)	

### Atomic displacement parameters (Å^2^)

**Table:**

	*U*^11^	*U*^22^	*U*^33^	*U*^12^	*U*^13^	*U*^23^
C1	0.0196 (10)	0.0311 (13)	0.0224 (12)	0.000	−0.0027 (9)	0.000
C2	0.0234 (11)	0.0525 (17)	0.0199 (12)	0.000	−0.0030 (9)	0.000
C3	0.0202 (11)	0.0525 (17)	0.0222 (12)	0.000	−0.0034 (9)	0.000
C4	0.0250 (9)	0.0422 (11)	0.0428 (11)	0.0061 (8)	−0.0008 (8)	0.0047 (9)
C5	0.0263 (9)	0.0474 (12)	0.0416 (11)	0.0037 (8)	0.0013 (8)	−0.0170 (9)
C6	0.0259 (8)	0.0350 (10)	0.0351 (10)	0.0003 (8)	−0.0028 (7)	−0.0077 (8)
C7	0.0273 (13)	0.082 (2)	0.0209 (13)	0.000	0.0009 (10)	0.000
C8	0.0222 (11)	0.0310 (13)	0.0216 (12)	0.000	−0.0039 (9)	0.000
C9	0.0265 (12)	0.0488 (17)	0.0216 (12)	0.000	−0.0056 (10)	0.000
C10	0.0243 (12)	0.0500 (17)	0.0252 (13)	0.000	−0.0064 (10)	0.000
C11	0.0211 (11)	0.0288 (13)	0.0216 (12)	0.000	−0.0003 (9)	0.000
C12	0.0224 (11)	0.0463 (16)	0.0247 (13)	0.000	−0.0024 (10)	0.000
C13	0.0255 (12)	0.0460 (16)	0.0228 (12)	0.000	−0.0024 (10)	0.000
C14	0.0426 (10)	0.0354 (10)	0.0317 (10)	0.0042 (9)	0.0069 (8)	0.0065 (8)
C15	0.0404 (11)	0.0464 (12)	0.0347 (11)	−0.0220 (9)	0.0052 (8)	−0.0050 (9)
C16	0.0387 (10)	0.0364 (11)	0.0314 (10)	−0.0119 (8)	0.0010 (8)	−0.0062 (8)
C17	0.0202 (12)	0.088 (3)	0.0292 (15)	0.000	−0.0004 (11)	0.000
N1	0.0209 (9)	0.0325 (11)	0.0223 (10)	0.000	−0.0026 (8)	0.000
N2	0.0208 (9)	0.0314 (11)	0.0224 (10)	0.000	−0.0030 (8)	0.000
Cl1	0.0333 (3)	0.0635 (5)	0.0279 (3)	0.000	−0.0021 (3)	0.000
Cl2	0.0435 (4)	0.0531 (4)	0.0281 (3)	0.000	0.0058 (3)	0.000
Cl3	0.0501 (3)	0.0395 (3)	0.0682 (4)	0.0181 (2)	0.0155 (3)	0.0136 (3)
Fe1	0.02563 (18)	0.0251 (2)	0.0268 (2)	0.000	0.00083 (14)	0.000

### Geometric parameters (Å, °)

**Table:**

C1—N1	1.499 (3)	C10—N2	1.383 (3)
C1—C2	1.520 (3)	C10—H10	0.9500
C1—C6	1.529 (2)	C11—N2	1.496 (3)
C1—C6^i^	1.529 (2)	C11—C16^i^	1.526 (2)
C2—C3	1.542 (3)	C11—C16	1.526 (2)
C2—H2A	0.9900	C11—C12	1.530 (3)
C2—H2B	0.9900	C12—C13	1.536 (3)
C3—C4	1.522 (2)	C12—H12A	0.9900
C3—C4^i^	1.522 (2)	C12—H12B	0.9900
C3—H3	1.0000	C13—C14	1.520 (2)
C4—C5	1.527 (3)	C13—C14^i^	1.520 (2)
C4—H4A	0.9900	C13—H13	1.0000
C4—H4B	0.9900	C14—C15	1.519 (3)
C5—C7	1.517 (3)	C14—H14A	0.9900
C5—C6	1.540 (2)	C14—H14B	0.9900
C5—H5	1.0000	C15—C17	1.526 (3)
C6—H6A	0.9900	C15—C16	1.537 (3)
C6—H6B	0.9900	C15—H15	1.0000
C7—C5^i^	1.517 (3)	C16—H16A	0.9900
C7—H7A	0.9900	C16—H16B	0.9900
C7—H7B	0.9900	C17—C15^i^	1.526 (3)
C8—N1	1.328 (3)	C17—H17A	0.9900
C8—N2	1.332 (3)	C17—H17B	0.9900
C8—H8	0.9500	Cl1—Fe1	2.1864 (7)
C9—C10	1.344 (4)	Cl2—Fe1	2.1830 (7)
C9—N1	1.376 (3)	Cl3—Fe1	2.1952 (6)
C9—H9	0.9500	Fe1—Cl3^i^	2.1952 (6)
			
N1—C1—C2	109.95 (19)	C16^i^—C11—C16	110.6 (2)
N1—C1—C6	108.24 (12)	N2—C11—C12	109.26 (17)
C2—C1—C6	109.97 (13)	C16^i^—C11—C12	109.45 (13)
N1—C1—C6^i^	108.24 (12)	C16—C11—C12	109.45 (13)
C2—C1—C6^i^	109.97 (13)	C11—C12—C13	109.04 (18)
C6—C1—C6^i^	110.4 (2)	C11—C12—H12A	109.9
C1—C2—C3	108.9 (2)	C13—C12—H12A	109.9
C1—C2—H2A	109.9	C11—C12—H12B	109.9
C3—C2—H2A	109.9	C13—C12—H12B	109.9
C1—C2—H2B	109.9	H12A—C12—H12B	108.3
C3—C2—H2B	109.9	C14—C13—C14^i^	110.2 (2)
H2A—C2—H2B	108.3	C14—C13—C12	109.17 (13)
C4—C3—C4^i^	109.9 (2)	C14^i^—C13—C12	109.17 (13)
C4—C3—C2	109.30 (13)	C14—C13—H13	109.4
C4^i^—C3—C2	109.30 (13)	C14^i^—C13—H13	109.4
C4—C3—H3	109.4	C12—C13—H13	109.4
C4^i^—C3—H3	109.4	C15—C14—C13	109.52 (16)
C2—C3—H3	109.4	C15—C14—H14A	109.8
C3—C4—C5	109.33 (16)	C13—C14—H14A	109.8
C3—C4—H4A	109.8	C15—C14—H14B	109.8
C5—C4—H4A	109.8	C13—C14—H14B	109.8
C3—C4—H4B	109.8	H14A—C14—H14B	108.2
C5—C4—H4B	109.8	C14—C15—C17	109.78 (17)
H4A—C4—H4B	108.3	C14—C15—C16	109.41 (16)
C7—C5—C4	109.56 (17)	C17—C15—C16	109.65 (19)
C7—C5—C6	109.68 (17)	C14—C15—H15	109.3
C4—C5—C6	109.52 (16)	C17—C15—H15	109.3
C7—C5—H5	109.4	C16—C15—H15	109.3
C4—C5—H5	109.4	C11—C16—C15	108.65 (15)
C6—C5—H5	109.4	C11—C16—H16A	110.0
C1—C6—C5	108.25 (16)	C15—C16—H16A	110.0
C1—C6—H6A	110.0	C11—C16—H16B	110.0
C5—C6—H6A	110.0	C15—C16—H16B	110.0
C1—C6—H6B	110.0	H16A—C16—H16B	108.3
C5—C6—H6B	110.0	C15^i^—C17—C15	109.3 (2)
H6A—C6—H6B	108.4	C15^i^—C17—H17A	109.8
C5^i^—C7—C5	110.0 (2)	C15—C17—H17A	109.8
C5^i^—C7—H7A	109.7	C15^i^—C17—H17B	109.8
C5—C7—H7A	109.7	C15—C17—H17B	109.8
C5^i^—C7—H7B	109.7	H17A—C17—H17B	108.3
C5—C7—H7B	109.7	C8—N1—C9	107.82 (19)
H7A—C7—H7B	108.2	C8—N1—C1	126.18 (19)
N1—C8—N2	109.6 (2)	C9—N1—C1	126.0 (2)
N1—C8—H8	125.2	C8—N2—C10	107.5 (2)
N2—C8—H8	125.2	C8—N2—C11	126.38 (19)
C10—C9—N1	107.7 (2)	C10—N2—C11	126.09 (19)
C10—C9—H9	126.2	Cl2—Fe1—Cl1	110.16 (3)
N1—C9—H9	126.2	Cl2—Fe1—Cl3^i^	109.40 (2)
C9—C10—N2	107.3 (2)	Cl1—Fe1—Cl3^i^	110.33 (2)
C9—C10—H10	126.3	Cl2—Fe1—Cl3	109.40 (2)
N2—C10—H10	126.3	Cl1—Fe1—Cl3	110.33 (2)
N2—C11—C16^i^	109.03 (12)	Cl3^i^—Fe1—Cl3	107.16 (3)
N2—C11—C16	109.03 (12)		
			
N1—C1—C2—C3	180.0	C16^i^—C11—C16—C15	−60.0 (2)
C6—C1—C2—C3	60.91 (14)	C12—C11—C16—C15	60.6 (2)
C6^i^—C1—C2—C3	−60.91 (14)	C14—C15—C16—C11	−60.7 (2)
C1—C2—C3—C4	−60.16 (14)	C17—C15—C16—C11	59.8 (2)
C1—C2—C3—C4^i^	60.16 (14)	C14—C15—C17—C15^i^	59.7 (3)
C4^i^—C3—C4—C5	−59.7 (2)	C16—C15—C17—C15^i^	−60.5 (3)
C2—C3—C4—C5	60.3 (2)	N2—C8—N1—C9	0.0
C3—C4—C5—C7	59.5 (2)	N2—C8—N1—C1	180.0
C3—C4—C5—C6	−60.9 (2)	C10—C9—N1—C8	0.0
N1—C1—C6—C5	178.92 (16)	C10—C9—N1—C1	180.0
C2—C1—C6—C5	−61.0 (2)	C2—C1—N1—C8	0.0
C6^i^—C1—C6—C5	60.6 (2)	C6—C1—N1—C8	120.14 (13)
C7—C5—C6—C1	−59.7 (2)	C6^i^—C1—N1—C8	−120.14 (13)
C4—C5—C6—C1	60.5 (2)	C2—C1—N1—C9	180.0
C4—C5—C7—C5^i^	−59.9 (3)	C6—C1—N1—C9	−59.86 (13)
C6—C5—C7—C5^i^	60.4 (2)	C6^i^—C1—N1—C9	59.86 (13)
N1—C9—C10—N2	0.0	N1—C8—N2—C10	0.0
N2—C11—C12—C13	180.0	N1—C8—N2—C11	180.0
C16^i^—C11—C12—C13	60.68 (13)	C9—C10—N2—C8	0.0
C16—C11—C12—C13	−60.68 (13)	C9—C10—N2—C11	180.0
C11—C12—C13—C14	60.27 (13)	C16^i^—C11—N2—C8	119.58 (13)
C11—C12—C13—C14^i^	−60.27 (13)	C16—C11—N2—C8	−119.58 (13)
C14^i^—C13—C14—C15	59.3 (2)	C12—C11—N2—C8	0.0
C12—C13—C14—C15	−60.6 (2)	C16^i^—C11—N2—C10	−60.42 (13)
C13—C14—C15—C17	−59.4 (2)	C16—C11—N2—C10	60.42 (13)
C13—C14—C15—C16	61.0 (2)	C12—C11—N2—C10	180.0
N2—C11—C16—C15	−179.91 (16)		

Symmetry codes: (i) *x*, −*y*+1/2, *z*.
